# Automated hand-marked semantic text recognition from photographs

**DOI:** 10.1038/s41598-023-41489-4

**Published:** 2023-08-30

**Authors:** Seungah Suh, Ghang Lee, Daeyoung Gil, Yonghan Kim

**Affiliations:** Department of Architecture and Architectural Engineering, Yonsei University, Seoul, 03722 Republic of Korea

**Keywords:** Engineering, Civil engineering

## Abstract

Automated text recognition techniques have made significant advancements; however, certain tasks still present challenges. This study is motivated by the need to automatically recognize hand-marked text on construction defect tags among millions of photographs. To address this challenge, we investigated three methods for automating hand-marked semantic text recognition (HMSTR)—a modified scene text recognition-based (STR) approach, a two-step HMSTR approach, and a lumped approach. The STR approach involves locating marked text using an object detection model and recognizing it using a competition-winning STR model. Similarly, the two-step HMSTR approach first localizes the marked text and then recognizes the semantic text using an image classification model. By contrast, the lumped approach performs both localization and identification of marked semantic text in a single step using object detection. Among these approaches, the two-step HMSTR approach achieved the highest F1 score (0.92) for recognizing circled text, followed by the STR approach (0.87) and the lumped approach (0.78). To validate the generalizability of the two-step HMSTR approach, subsequent experiments were conducted using check-marked text, resulting in an F1 score of 0.88. Although the proposed methods have been tested specifically with tags, they can be extended to recognize marked text in reports or books.

## Introduction

Automated text recognition plays a crucial role in processing paper documents^1^ and detecting textual information in various image types, such as road signs and billboards in natural scenes^2,3^, handwritten notes^4^, and identifiers in diagrams^5^. Among the various text recognition tasks, this study focuses on automated hand-marked semantic text recognition (HMSTR) from photographs. Hand-marked semantic text refers to "semantic text" in an image that has been marked with a hand-drawn circle or checkmark to indicate its importance or selection. Semantic text generally consists of a string of characters that conveys semantic information^6^. For instance, "kitchen" is considered semantic text, whereas "lorem ipsum" is not. However, semantic text is not limited to general words or phrases: it can include symbols, numbers, or abbreviations that hold specific meanings depending on the context. For example, in the context of the movie Star Wars, "R2D2" holds a significant meaning as the model name of a robot. Similarly, in an architectural context, "R2" and "D2" may indicate "Bedroom 1" and "Dining Room 2," respectively.

**Figure 1:**
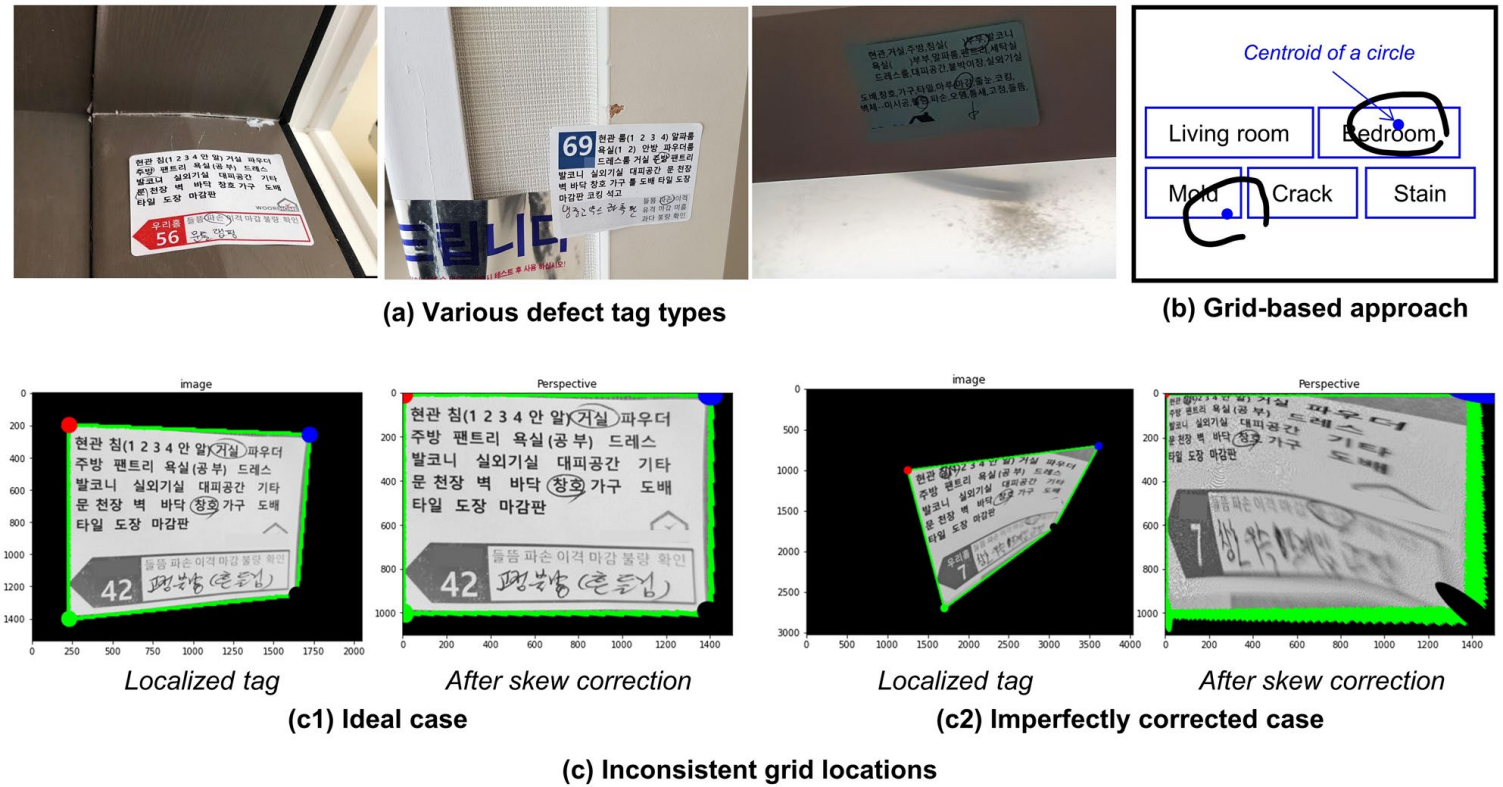
Basic concept and problems of a grid-based method.

The motivation for this study stems from the need to automatically identify hand-marked semantic text information in millions of construction defect photographs. These photographs typically include a defect tag placed next to a defect, explicitly indicating the defect's details such as its type and location (Fig. 1a). It is important to note that the proposed methods are not limited to this specific scenario but can be applied to various other situations, such as recognizing marked text in a photograph of a safety tag attached to a problematic area; a photograph of an invoice tag hung on delivered materials and products like pipes; hand-marked keywords or phrases in academic papers, books, or diagrams; marked corrections in manuscripts; hand-marked survey questionnaires; and medical or mechanical checklists.

The problem at hand falls within the domain of optical character recognition (OCR), which includes tasks involving the recognition of handwritten text^7^ and scanned documents with^8^ or without occluded text^9^. Among the various OCR tasks, scene text recognition (STR)—recognizing text in images of complex scenes such as speed limits in cityscape photographs—comes closest to HMSTR. STR typically involves detecting text areas at the word or character level using object detection and instance segmentation, and the model then recognizes the text based on the extracted features from the network^10,11^. Unlike the classification of Modified National Institute of Standards and Technology (MNIST) dataset images^12^, STR recognizes handwritten digits and printed letters positioned in varying real-world conditions. STR models implement additional measures such as powerful image normalization and sequence modeling to adjust to the complicated conditions. Recent advancements in STR have focused on developing context-aware methods to improve text recognition accuracy. For instance, He et al.^13^ proposed a segmentation baseline with graph-based textual reasoning (S-GTR), which utilizes a graph convolutional network to incorporate visual context for textual reasoning. Bautista and Atienza^14^ enhanced STR performance by combining context-free and context-aware autoregressive inference.

**Figure 2:**
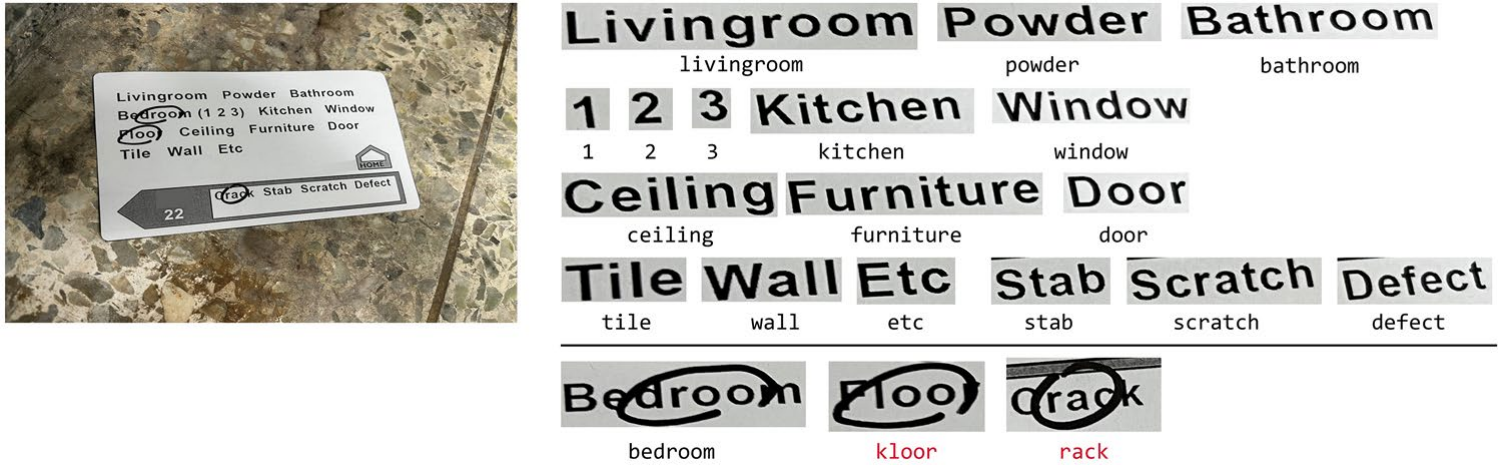
Examples of failed hand-marked text recognition using a state-of-the-art optical character recognition model.

However, even a state-of-the-art STR model^13^ struggles with HMSTR owing to the presence of irregular hand-drawn markers, adding complexity to the localization and recognition of the text. This difficulty arises from several factors. First, the hand-marked semantic text is often obscured by randomly drawn markers that vary in shape, thickness, and color, as illustrated in Figs. 1 and 2. Second, hand-drawn markers can be mistaken for strokes of letters, as demonstrated by the "kloor (floor)" example in Fig. 2, leading to misinterpretation. Third, the ambiguity of the boundary of a circle sometimes results in only a portion of a word being recognized, as seen in the "rack (crack)" example in Fig. 2. Fourth, HMSTR involves the selective extraction of only the "marked" words, rather than recognizing all words in an image like typical OCR models^15^. Fifth, hand-marked texts often lack contextual information, as depicted in Fig. 2. Consequently, context-aware STR approaches are not applicable. Finally, as shown in Fig. 1, texts in photographs are subject to distortion and skewing, presenting challenges even for human readers.

To address these challenges, an initial grid-based approach was considered. This approach involves drawing invisible grid lines along the semantic texts in an image, with each grid cell storing the corresponding semantic text information. For example, the first grid cell contains the text "Living room." The approach then detects the centroid of a hand-drawn mark using object detection and determines the grid cell in which the centroid falls. The semantic text associated with that grid cell is considered to be the marked semantic text. Figure 1b provides an illustration of the fundamental concepts of this approach. In the figure, the centroids of the hand-drawn circles fall into the second and third grid cells, resulting in the identification of the marked semantic texts as "Bedroom" and "Mold".

However, the grid-based approach has two major limitations. First, it is effective only when a standardized template is used across all collected cases, whereas in practice, each case differs. For instance, various defect tag designs are used for different projects, as shown in Fig. 1a. Second, the grid-based approach performs poorly when dealing with arbitrarily photographed images. In an ideal scenario, skewed images can be almost perfectly straightened through skew correction, as demonstrated by the example on the left in Fig. 1c. However, in many cases, even after skew correction, skewed images do not form clean vertical and horizontal grid lines, as shown in the example on the right in Fig. 1c. In the case of defect tags, they are photographed near the actual defect to accurately indicate its location and associated information. Consequently, defect tags often appear as artifacts in photographs rather than as clear scanned images. It is challenging to reconstruct the intended grid lines from such images, rendering the grid-based approach ineffective.

**Figure 3:**
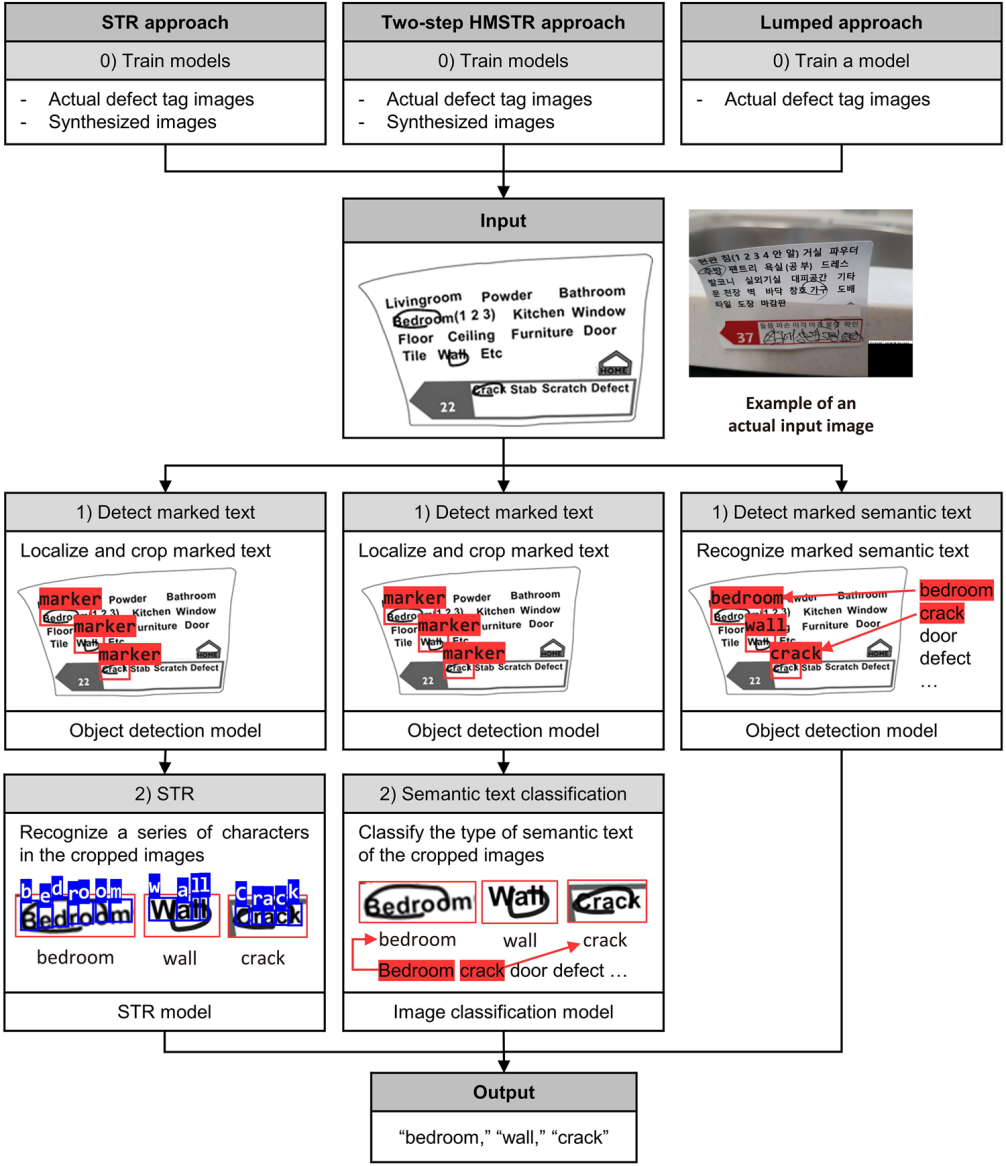
High-level conceptual comparison of three approaches.

This study proposes three methods to address these challenges: a modified STR-based approach (referred to as the STR approach), a two-step HMSTR approach, and a lumped approach, as illustrated in Fig. 3. The STR approach utilizes an object detection model to localize only the marked texts and recognizes the characters within the detected region using an STR model. Similarly, the two-step HMSTR approach localizes the marked texts, crops the identified regions using an object detection model, and employs an image classification model to recognize text at the semantic text level instead of the character level. The lumped approach combines localization and identification of marked semantic texts in a single step using object detection. Although post-correction techniques^16,17^ can further improve the performance of the proposed methods, this study focuses solely on enhancing HMSTR performance without post-correction.

The performances of the three proposed methods were compared and tested in a series of experiments involving the recognition of marked defect information on defect tags collected from South Korean apartment construction projects. The first set of experiments analyzed the F1 score of the three methods to determine the best-performing approach using images of "circled text" in construction defect tag photographs. Subsequently, as the first set of experiments focused on "circled text," the second set of experiments evaluated the generalizability of the proposed method by conducting tests on "check-marked text," verifying whether the proposed method can be applied to other types of markers.

## Three approaches

### STR approach

The STR approach, although referred to as such, is not a simple fine-tuned version of an existing STR model. It consists of two distinct steps: fine-tuning an object detection model to isolate marked text and fine-tuning an STR model to identify characters within the isolated images^18^. Transfer learning, a concept that involves transferring knowledge from a related source to a target task, is employed^19^. Fine-tuning, which is a method of transfer learning, begins with a pretrained model on the source task and further trains it on the target task by unfreezing the whole neural network layers^19^. In the case of the STR approach, the detection model pretrained on the COCO dataset^20^ is fine-tuned to localize markers and their corresponding semantic text using real-world images of marked text. The model detects marked text within the entire image and defines bounding boxes for each instance of marked text. Subsequently, the image is cropped along the bounding boxes. An existing STR model, which is fine-tuned on the marked semantic text and possibly other languages, extracts text from the cropped images. Similar to typical STR models, the STR approach does not recognize character sequences as meaningful expressions but identifies each individual character in the image and combines them^10^.

**Figure 4:**
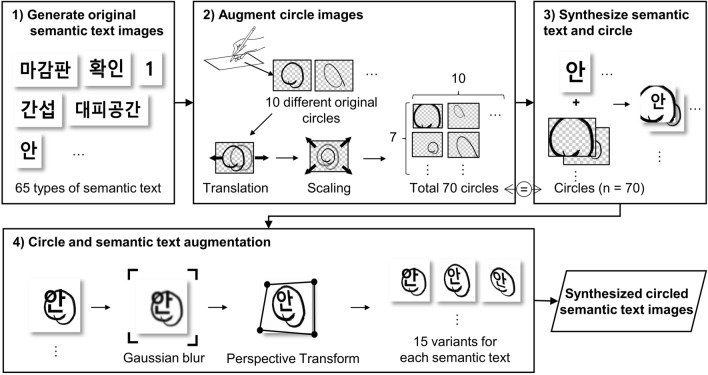
Process of image synthesis and augmentation.

While training the STR model requires a sufficient number of marked text images, the actual availability of such images is often limited, making it necessary to generate synthetic images. Synthetic images are created by overlaying multiple marker shapes on unmarked text images and subsequently augmented in a rule-based manner to cover a wider range of cases. Figure 4 illustrates the four stages of image synthesis and augmentation. First, original semantic text images were created for all 65 types of semantic texts. Second, digitized original circle images were translated and scaled in the second stage to create seven variants for each circle. The translation factor was randomly defined between 20% of the width and height of each image. The gap change was 0.05% in width and height. Similarly, the scaling factor was randomly chosen from 0.5 to 1.5 with a change gap of 1, generating 70 variants of circles. Third, the augmented circle images were overlaid on the text images, resulting in 70 types of circled words for every 65 semantic texts. Finally, circled text images were augmented using Gaussian blur and a perspective transformation. Gaussian blur, also known as Gaussian filter, reinforces contrast and restrains speckles, making the edges and borders of images hazy^21^. Three iterations of Gaussian blur were applied to the images, and its blur kernel size was set as (5, 5) with a sigma ranging from 0.1 to 10. The change gap was set as 0.10. The augmented and original images were perspective-transformed over five iterations. Perspective transformation is defined as a distortion owing to the projection of objects onto a 2D image plane at different distances to the camera^22^. The distortion scale was 0.5, with a probability of 1, and the change gap of the target coordinate was 1.

### Two-step hand-marked semantic text recognition (HMSTR) approach

The two-step HMSTR approach stems from the idea that recognition accuracy would improve if marked text was identified at the semantic text level rather than at the character level. This approach begins by utilizing an object detection model that is fine-tuned to isolate the marked text in an image, similar to the STR approach. It determines the bounding box for each marked text and crops the identified regions. Subsequently, an image classification model is employed to recognize the text at the semantic text level, which differs from typical STR models. The image classification model is transfer-learned by adding fully connected layers to recognize the meaning of the identified text. Technically, the model does not "read" the text but rather categorizes a cropped marked text image as one of the possible semantic texts from a predefined set of semantic text categories. This set varies depending on the target document and includes all terms of interest. Rule-based image synthesis and augmentation are also employed to generate additional marked semantic text images for training.

### Lumped approach

The lumped approach is designed with a straightforward single-step structure to ensure its applicability. It utilizes a fine-tuned object detection model to localize marked semantic text within an entire image and classifies its type in a single step based on a predefined set of semantic text categories^19^. To fine-tune the object detection model, only full images with annotated marked semantic text are required for training. The lumped approach relies solely on real-world images for fine-tuning to prevent models from overfitting to unrealistic full tag images^23^. Although other methods can benefit from rule-based image synthesis when only "marked text" images are needed, generating complete images for the lumped approach is more challenging as they should encompass the entire marked tag along with a cluttered background. Consequently, there is a risk that rule-based image synthesis may generate overly simplistic or excessively distorted images that deviate significantly from the actual pictures. Alternatively, the possibility of using generative adversarial networks (GANs) for image synthesis was considered^24^. However, GANs did not perform well in creating images with accurately represented text, and exploring their potential in this context could be a separate research topic.

## Results and discussion

### First set of experiments

Table 1 summarizes the results obtained from the first set of experiments, including overall mean precision, recall, F1 score, and weighted F1 score values. The approach with the highest F1 score, which is the harmonic mean of precision and recall^25^, was considered the most accurate. The weighted F1 score was also provided to evaluate the proposed methods, considering class imbalance^26^. However, the F1 score was chosen as the main evaluation metric because recognizing infrequently occurring defects is equally, if not more, important than identifying frequently occurring defects. The dataset used for evaluation consisted of a total of 65 Korean semantic texts, as listed in Table 2.

**Table 1:** Performance of three methods.

	STR approach			Two-step HMSTR approach			Lumped approach
	Circled text detection	STR	Overall	Circled text detection	Semantic text classification	Overall	Circled semantic text detection
Precision	0.97	0.89	0.86	0.97	0.95	0.92	0.94
Recall	0.99	0.89	0.88	0.99	0.93	0.92	0.77
F1 score	0.98	0.89	0.87	0.98	0.94	0.92	0.78
Weighted F1 score	0.98	0.70	0.69	0.98	0.98	0.96	0.94

**Table 2:** Types of semantic text.

No.	Semantic text in English	No.	Semantic text in English	No.	Semantic text in English
1	1	22	Dress room	44	Lock
2	2	23	Electrical outlet	45	Loose
3	3	24	Etc	46	Main
4	4	25	Evacuation space	47	Missed
5	Alpha	26	Excessive	48	Mold
6	Balcony	27	Finishing	49	Off-set
7	Bathroom	28	Finishing plate	50	Omission
8	Bedroom	29	Fixed	51	Open
9	Burnt	30	Floor	52	Operation
10	Calking	31	Front door	53	Painting
11	Ceiling	32	Furniture	54	Pantry
12	Check	33	Gap	55	Papering
13	Closet	34	Horizontal and vertical	56	Plant room
14	Connection	35	In bedroom	57	Plastering
15	Crack	36	In living room	58	Pollution
16	Damaged	37	Insufficient	59	Powder room
17	Defect	38	Interference	60	Scratch
18	Defect on face	39	Joint	61	Sealing
19	Detached	40	Kitchen	62	Stab
20	Detail	41	Laundry room	63	Tile
21	Door	42	Lighting	64	Wall
		43	Living room	65	Window

The F1 scores for the STR, two-step HMSTR, and lumped approaches were 0.87, 0.92, and 0.78, respectively. Similarly, the weighted F1 scores for the STR, two-step HMSTR, and lumped approaches were 0.69, 0.96, and 0.94, respectively. In terms of both evaluation metrics, the two-step HMSTR approach demonstrated the best performance compared with the other two approaches.

The STR and the two-step HMSTR approaches are composed of two common steps: marked text detection and text recognition. Thus, the difference in performance between the two approaches can be attributed to their contrasting text recognition capabilities. The STR approach, which treats marked text as a sequence of characters, performed poorly (F1 score of 0.89) than the two-step HMSTR approach (F1 score of 0.94), which recognizes semantic text as a single entity.

In contrast to the other two approaches, the lumped approach exhibited a significant performance gap between the F1 score and the weighted F1 score. It achieved the lowest F1 score of 0.78, while its weighted F1 score was relatively high (0.94). This indicates that the lumped approach performs well in recognizing frequently occurring semantic texts but struggles with infrequently occurring ones. To improve the F1 score of the lumped approach, generating synthetic images of infrequent cases for retraining the model could be beneficial. However, as explained in the "Three approaches" section, the generation process presents challenges.

### Second set of experiments

To assess the generalizability of the two-step HMSTR approach, which performed the best, additional experiments were conducted using defect tag photographs with check-marked text. Table 3 presents the performance results, indicating that the two-step HMSTR approach performed well in recognizing check-marked text, achieving an F1 score of 0.88 and a weighted F1 score of 0.94. Specifically, the F1 score for check-marked text detection was 1.0, which is higher than that for circled text detection. However, the F1 score for semantic text classification of check-marked semantic text was lower compared with circled semantic text, possibly due to the relatively unclear boundary of the check-marked text.

**Table 3:** Performance of the two-step HMSTR approach with circled and check-marked texts.

	Two-step HMSTR with circled texts			Two-step HMSTR with check-marked texts		
	Detection	Semantic text classification	Overall	Detection	Semantic text classification	Overall
Precision	0.97	0.95	0.92	1.0	0.89	0.89
Recall	0.99	0.93	0.92	1.0	0.89	0.89
F1 score	0.98	0.94	0.92	1.0	0.88	0.88
Weighted F1 score	0.98	0.98	0.96	1.0	0.94	0.94

## Limitations and future works

Although the two sets of experiments confirmed the effectiveness of the two-step HMSTR approach in recognizing hand-marked text in photographs, there are still limitations to address. First, the two-step HMSTR approach can only identify a predefined set of semantic texts, unlike the STR, which can handle infinite combinations of characters. Despite this limitation, the applicability of the proposed approach is not excessively restricted, as it can cover the desired semantic texts by adding them to the predefined list as necessary. For example, the test dataset already included 65 classes of semantic texts, and incorporating additional semantic texts into the list is not challenging. Furthermore, the two-step HMSTR approach offers an advantage in scenarios wherein a photograph contains a mix of texts of interest and no interest. By restricting the selection to the predefined list of semantic texts, the two-step HMSTR approach can easily differentiate the semantic texts of interest from the others. This becomes particularly useful as texts of interest are often limited to real-world problems, rendering the two-step HMSTR approach effective in most scenarios. However, if the target texts are unknown, the STR approach would be more suitable, despite potentially lower performance when compared with that of the two-step HMSTR approach.

Second, the performance of the two-step HMSTR approach needs further validation using different languages and models. The method was evaluated using only Korean. Nevertheless, it is expected that the proposed approach would perform better in more commonly studied languages such as English because processing Korean text is generally more challenging due to its unique way of composing one syllable^27^. The proposed method is also expected to perform well on pictographic text because it treats each marked text as an image.

Finally, a different approach may prove more effective than the proposed method in a digitalized environment. However, recognizing hand-marked text remains a significant area of study, as analog systems employing paper and pencils continue to be widely utilized daily, alongside the persistent issue of legacy data.

Future studies will include experiments using other languages and models. The proposed method can be extended to more complex settings involving pictographic text and mixed marker shapes with different meanings. It can also be validated with a larger set of semantic texts and other types of images containing marked semantic text.

## Conclusion

Although marking words is a common practice to emphasize certain information in a document, accurately recognizing marked texts in photographs remains a challenge for existing methods. This study proposes three novel approaches: STR, two-step HMSTR, and lumped, aimed at enhancing the performance of hand-marked semantic text recognition. To compare the effectiveness of these approaches, we conducted experiments using Korean defect tag images with hand-drawn circles. Among the three methods, the two-step HMSTR approach achieved the highest F1 score (0.92) and weighted F1 score (0.96). Furthermore, we tested the generalizability of the two-step HMSTR approach on Korean defect tag images with check-marked text, resulting in an F1 score of 0.88 and a weighted F1 score of 0.94.

This study makes a significant contribution by addressing the unexplored but practical HMSTR task, which cannot be effectively handled by state-of-the-art OCR and STR techniques^13^. Specifically, our experiments demonstrate that the proposed approach effectively recognizes hand-marked domain-specific terms, jargon, loan words, and abbreviations^28^, for which other typical text recognition models lack training. Moreover, the two-step HMSTR approach is expected to reduce the time and effort required to extract valuable information from various data sources. These sources include photographs of safety inspection tags attached close to dangerous areas, photographs of invoice tags loosely hung on products, and general images of hand-marked text in documents.

## Experimental methods

The three proposed methods’ performance was tested in a series of experiments employing South Korean construction defect tag images that contain hand-marked semantic texts. The following subsections describe the datasets and experimental methods used in detail. The essential parts of the codes for the experiments can be retrieved from the following link: https://github.com/Suh10000/Two-step-HMSTR.git.

### Dataset

**Figure 5:**
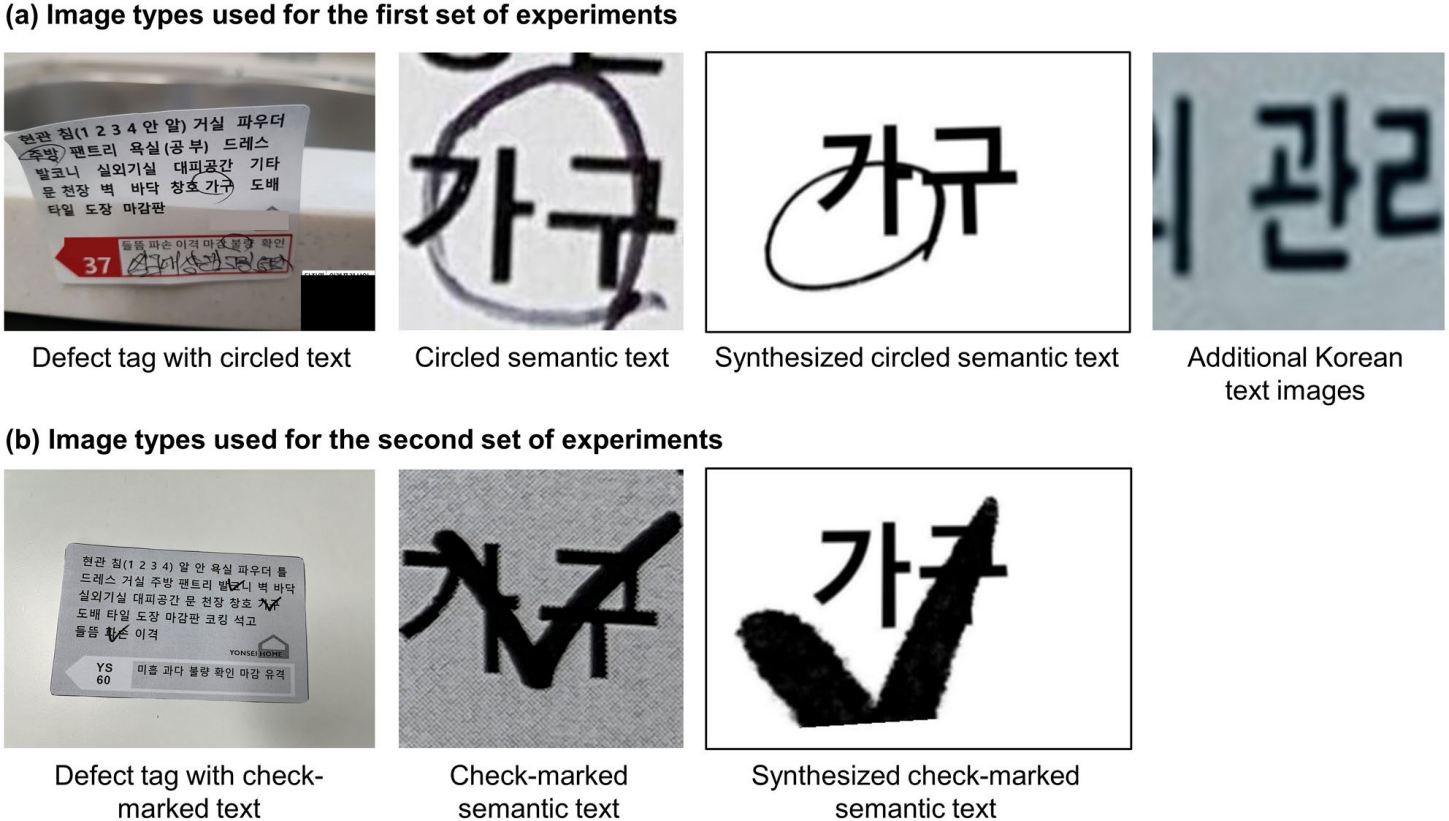
Sample images for each image type.

Figure 5a and b depict the types of images used for the first set and second set of experiments, respectively. Table 4 summarizes the exact amount of data used. In both experiments, defect tag images and hand-marked semantic text images were employed. A total of 65 Korean semantic texts, as presented in Table 2, appeared in 11 types of defect tags. Each type of defect tag contained a different combination of around 30 semantic texts and possessed a unique text layout, shape, and color, as shown in Fig. 1a. The circles and corresponding semantic text were manually annotated to train the models. Bounding boxes were labeled on the 2,046 original images to include markers and the corresponding semantic text. According to the annotation, the original images were cropped, resulting in 6,770 semantic text images for the experiments.

**Table 4:** Data description of the two sets of experiments.

Method	Task	Image type	Train	Validation	Test	Total
The first set of experiments						
STR approach	Circled text detection	Defect tag with circled text	1577	429	40	2046
	STR	Circled semantic text	3434	2003	1333	6770
		Synthesized circled semantic text	70,493	15,957	0	86,450
		Additional Korean text for fine-tuning	14,901	1946	0	16,847
		Total images used for STR	88,828	19,906	1333	110,067
Two-step HMSTR approach	Circled text detection	Defect tag with circled text	1,577	429	40	2046
	Semantic text classification	Circled semantic text	3434	2003	1333	6770
		Synthesized circled semantic text	70,493	15,957	0	86,450
Lumped approach	Circled semantic text detection	Total images used for semantic text classification	73,927	17,960	1333	93,220
The second set of experiments						
Two-step HMSTR approach	Marked text detection	Defect tag with check-marked text	160	20	20	200
	Semantic text classification	Check-marked semantic text	233	110	62	405
		Synthesized check-marked semantic text	70,493	15,957	0	86,450

**Figure 6:**
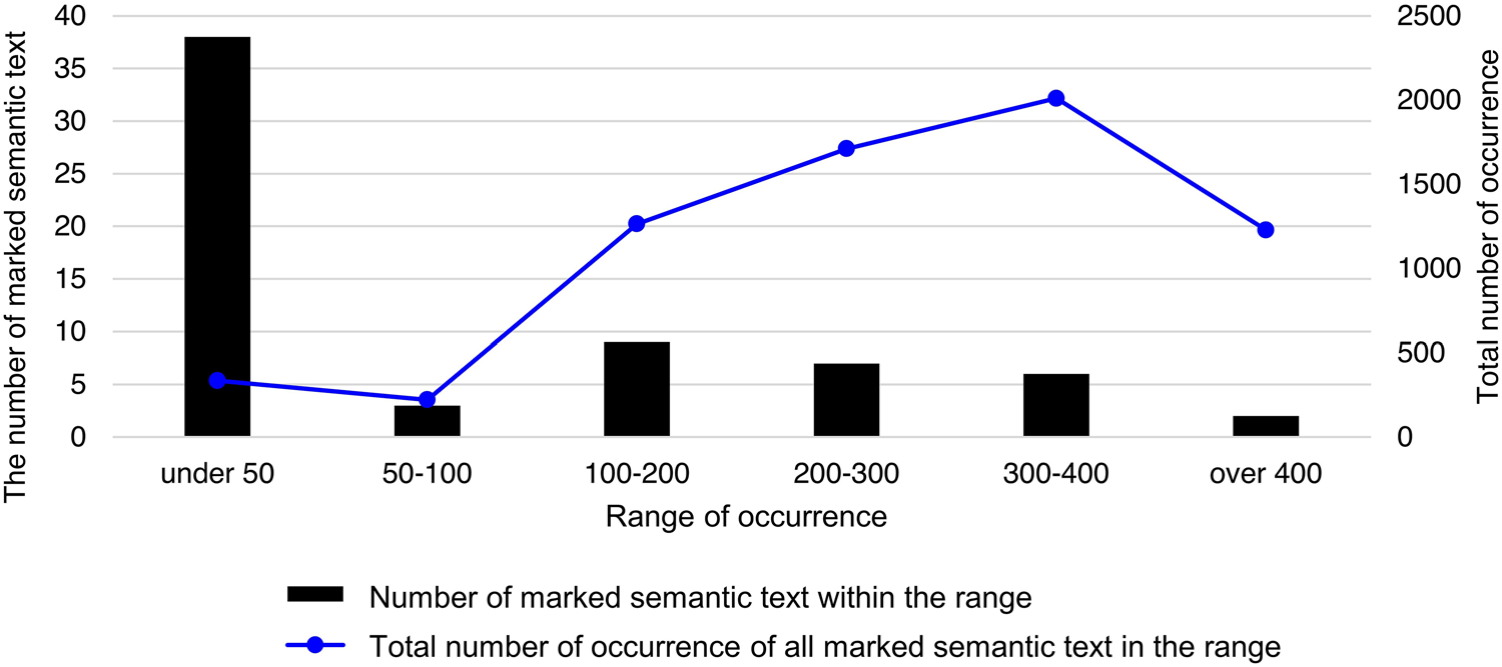
Data composition of cropped circled semantic text.

As explained previously, the STR and two-step HMSTR approaches leverage image synthesis and augmentation techniques to supplement imbalanced data^29^. Figure 6 provides an overview of the manner in which a few types of marked semantic text account for most occurrences in the dataset. Following the entire image synthesis and augmentation process as explained in the “Three approaches” section, 86,450 synthesized images were generated.

As the experiments used Korean text, additional Korean text images were deployed to fine-tune the STR model for Korean. Additional Korean text images were acquired from AI Hub^30^, which is a South Korean national platform disclosing high-quality and high-capacity data for artificial intelligence research and development^31^. The Korean text image dataset contained printed text, handwritten text, and text in real-world scenarios. The first two datasets were artificially generated, whereas the real-world text corresponded to a dataset of real-world images. Among the various images provided by AI Hub, book cover images, a subset of real-world text in the Korean text image dataset, were employed for training. Text in the dataset encompassing real-world scenarios is classified into four categories: book covers, goods, signboards, and traffic-sign images^31^. Among them, book cover images were selected to train the STR model, as other categories often have unique fonts or vivid colors, unlike defect tags. As AI Hub annotates all Korean words in the dataset, the original book cover images were cropped based on the given bounding box information. Consequently, 16,847 Korean text images were obtained and used to train the STR model in Korean.

### First set of experiments

**Figure 7:**
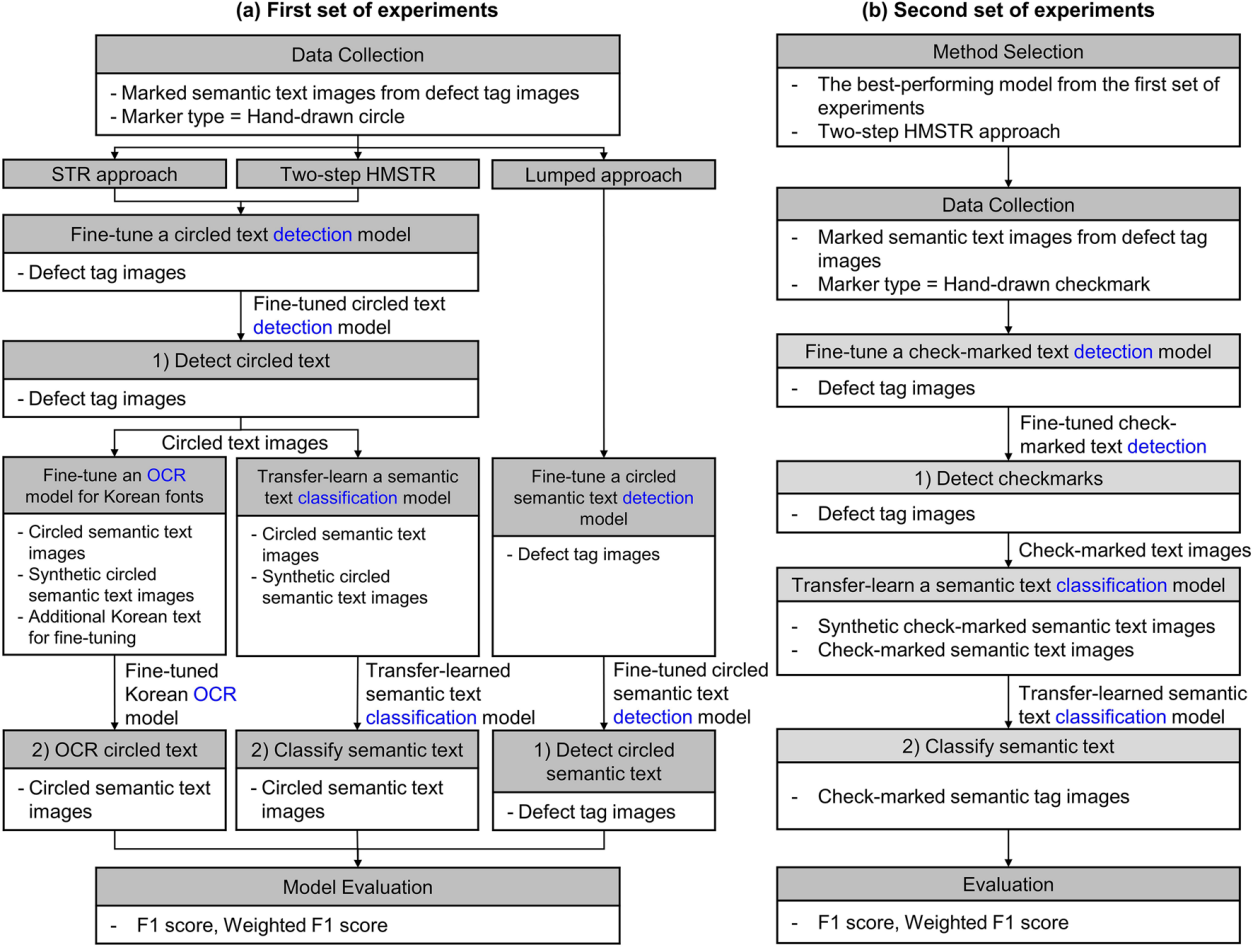
Flow of the first and second set of experiments.

The initial set of experiments aimed to identify circled text in defect tag photographs. Figure 7a provides an overview of the research flow for this set of experiments. First, a total of 2,046 defect tag images containing 6770 circled semantic texts were collected. Second, the STR model by Baek et al.^10^, You Only Look Once version 5 (YOLOv5)^32^, and Vision Transformer (ViT)^33^ were trained on marked text images as the STR, object detection, and object classification models, respectively. The choice of these models is justified as follows.

For the STR approach, the STR model developed by Baek et al.^10^ was fine-tuned specifically for Korean-marked text owing to the data being in Korean. This model had previously won first prize in the multi-language script recognition task at the prestigious International Conference on Document Analysis and Recognition (ICDAR) 2019 Robust Reading Challenge on Arbitrary-Shaped Text (RRC-ArT)^34^. The model consists of four stages commonly found in STR models: transformation, feature extraction, sequence modeling, and prediction. It offers various options for each stage. In these experiments, thin-place spline (TPS)^3,35^ transformation was applied for the transformation stage, residual net (ResNet)^36^ for feature extraction, bidirectional long short-term memory (BiLSTM)^37^ for sequence modeling, and attention-based sequence prediction (Attn)^3,38^ for prediction, considering their accuracy^10,39^. The fine-tuning process utilized 5437 circled semantic text images (3434 for training and 2003 for validation) and 86,450 synthetic marked text images (70,493 for training and 15,957 for validation). Additionally, 16,847 Korean font images (14,901 for training and 1946 for validation) were employed to fine-tune the model specifically for Korean. The training was iterated 8000 times, and the model was tested using the remaining 1333 marked semantic text images.

All three approaches employed fine-tuned object detection models to extract or localize marked text from the original images using 2006 defect tag images (1577 for training and 429 for validation). While several state-of-the-art (SOTA) object detection models, including EfficientNet, Single Shot MultiBox Detector (SDD), and faster region-based convolutional neural network (Faster R-CNN) algorithms, were considered, YOLOv5 was chosen based on a comparative analysis study by Rocha et al.^40^. YOLOv5 is known for its lightweight and fast performance while achieving SOTA results. It also offers input data enhancement, such as random image size processing, color space adjustment, and mosaic data augmentation^41^, which aids in detecting small objects by combining multiple images^42^. The training of the YOLOv5 model employed an image size of 416 × 416 pixels, a batch size of 32, and 300 epochs. During the prediction stage, the confidence threshold value for YOLOv5 was set to 0.45, determined through experiments starting from 0.5^43^. The remaining 40 defect tag images were used for testing the models.

ViT was chosen for the image classification task based on the comparable efficiency of ViT models to SOTA convolutional neural networks validated by Gheflati and Rivaz^44^. Additionally, Han et al.^45^ demonstrated that ViT achieved SOTA performance for handling small data points when pretrained on large datasets containing over 14 million images. The model used in these experiments was pretrained on ImageNet-21K, fulfilling the mentioned conditions^46^. The pretrained ViT model was transfer-learned to classify cropped circled images into 65 categories of semantic text. The extracted features from the pretrained ViT model were flattened and fed into a dense layer with 11 units. Finally, a dense layer with 65 units classified the category of the input images. The image resolution, batch size, and iteration epochs were set to 224 × 224 pixels, 32, and 11, respectively. These hyperparameters were optimized heuristically. The same dataset used to train the STR model, excluding the additional Korean text images, was used for training and evaluating ViT.

Ultimately, the F1 and weighted F1 scores of each approach were compared to determine the best-performing method. The final score of an approach is calculated by multiplying the corresponding scores of each stage, indicating the rate of correctly identified marked text images to the total number of images.

### Second set of experiments

In the second set of experiments, actual defect tag images with hand-drawn checkmarks were used to test whether the best-performing approach worked on different marker shapes other than circles. Figure 7b illustrates the overall experimental process. First, the best-performing model was selected based on the results of the first set of experiments. Second, 200 defect tag images with check-marked text were collected, and from these images, 405 actual check-marked semantic text images were obtained. The number of semantic texts was 65, identical to the first set of experiments. Third, an object detection model was fine-tuned for check-marked text using 180 (160 for training and 20 for validation) defect tag images. The same models and training conditions as those used in the first set of experiments were applied in the second set of experiments. The only differences were the dataset and the task of identifying check-marked semantic texts. The model was then tested on the 20 remaining images. Subsequently, an image classification model was transfer-learned to semantic text images. The training employed 343 (233 for training and 110 for validation) actual check-marked semantic text images and 86,450 synthetic check-marked semantic text images. The entire image synthesis and augmentation process leveraged in the second set was identical to that in the first set, except for the marker shape. Subsequently, the model was tested using 62 actual check-marked text images. Finally, the generalizability of the two-step HMSTR approach was evaluated using the F1 score and weighted F1 score.

## Data Availability

The data that support the findings of this study are available from the third party, but restrictions apply to the availability. The data were used under license for the current study and are not publicly available. Data can be available from the authors upon reasonable request and with permission of the third party.
